# The stakeholder model: its relevance, concept, and application in the Indonesian banking sector

**DOI:** 10.1057/s41261-020-00140-2

**Published:** 2021-01-06

**Authors:** Yafet Yosafet Wilben Rissy

**Affiliations:** Faculty of Law, Satya Wacana Christian University, Salatiga, Indonesia

**Keywords:** Indonesian banking sector, IFSA, Stakeholder, Normative principles, Practical principles

## Abstract

This article examines the current Indonesian Financial Service Authority (IFSA) regulations on corporate governance that deal with the relevance, concept, and application of the stakeholder model in the Indonesian banking sector. This study shows that the current IFSA regulations on corporate governance in the Indonesian banking sector encourage the application of the stakeholder model. However, they contain a vague definition of a stakeholder, fail to properly identify the legitimate stakeholders of the Indonesian banking sector, and provide no principles that can be used to align bank stakeholders’ interests. IFSA should revise these regulations so that they are more compatible with the theoretical basis and international best practices. This can be done through providing a concise definition of the concept of a stakeholder and offering normative and practical principles to be used when identifying the Indonesian banking sector legitimate stakeholders and aligning stakeholders’ interests.

## Introduction

The stakeholder model was formulated over three decades ago. Despite the model’s broad acceptance, questions still arise as to how it might be implemented in practice, especially in countries such as Indonesia, where the stakeholder model is still in its infancy including in the Indonesian banking sector. The introduction of the stakeholder model in the Indonesian banking sector can be seen in the Indonesian Financial Services Authority (IFSA) regulations concerning corporate governance for both general banks (2016) and PCBs (2015). In these two main regulations, it is simply stipulated that the Indonesian banking sector should implement good corporate governance to protect its stakeholder (consideration letter b). The Indonesian banking stakeholders are those who have direct or indirect stakes with a bank’s business operations, and the Indonesian banking sector should disclose its financial reports to its stakeholders periodically (Article 1 (8) of IFSA Corporate Governance Regulation for General Banks (2016) and for PCBs (2015)). These prevailing rules indicate that IFSA should simply oblige the Indonesian banking sector to implement the stakeholder model to protect its legitimate stakeholders and to achieve its long-term success or value creation.

It is to be acknowledged that the introduction of the stakeholder model in the Indonesian banking sector is a strategic step to alter the industry’s management considering the fact that the industry has been long managed under a strong bias shareholder primacy culture that led to 1999 financial crisis and the closure of a number of banks before, during, and after 1999 crisis [20, 36, 60, 70, 83, 85, 86, 88]. However, the relevance, concept, and application of the stakeholder model in the Indonesian banking sector are still unclear. There are three main critical issues which remain to be examined. They are the unclear concept of stakeholders in the regulations, the failure of the regulations to properly identify the legitimate stakeholders of the Indonesian banking sector, and the lack of principles that can be used to align the Indonesian banking sector stakeholders’ interests.

Several studies have been conducted in relation to the concept and application of the stakeholder model in broader companies in Indonesia. Spitzeck and Hansen [89], for example, investigated the influence of stakeholders in corporate decision-making in the Tangguh Liquefied Natural Gas Project. They found that both the mass traditional customer integration mechanism and conventional stakeholder dialogues and advisory board contributed significantly to the corporate business decisions. Ramadhini et al. [84] studied the effects of external stakeholder pressure and corporate social responsibility (CSR) disclosure. They revealed there is a positive correlation between the pressures of the external stakeholders, such as creditors, media exposure, and environmental disclosure. Another study focused on the effects of stakeholder and corporate governance pressures on the quality of sustainability reports, as discovered by Rudyanto and Siregar [87], where they insisted that companies that have environmental and consumer pressures have much better sustainability reports than those that do not have the same pressures.

In the context of the stakeholder model and banking sector, Manaf and Suryadi [68] found that in the debt dispute between Bank Dagang Nasional Indonesia (BDNI) and the Indonesian Bank Restructuring Agency (IBRA), there were direct and indirect influences of stakeholders in mitigating the release of BDNI’s debt. Furthermore, in the field of PCBs, it must be recognized that various research has been conducted by Duetsche Gesellschaft Technische Zusammenarbeit and Bank Indonesia [26], Duetsche Gesellschaft Technische Zusammenarbeit [27], Lapenu [65], Meagher et al. [67], Rissy [85], and Rissy [86]. Mainly, they focused on PCBs’ financial and organizational regulatory frameworks as microfinance institutions and corporate governance standards in PCBs.

Nevertheless, to this point, it is obvious that none of the previous studies dealt with the relevance, concept, and application of the stakeholder model in the Indonesian banking sector. This study attempts to fill this gap by examining and critiquing recent developments in the field, investigating the concept and application of the stakeholder model in the Indonesian banking sector, and providing recommendations to make its application more consistent with the theoretical and international best practice perspectives.

This article begins by explaining the theoretical background of the stakeholder model. It is then followed by an explanation of the relevance of the stakeholder model for the Indonesian banking sector as well as an examination of the current IFSA Regulations on Corporate Governance which focuses on the flaws in the existing regulations. This examination deals with three fundamental issues. They are the unclear definition of a stakeholder, the identification of legitimate stakeholders in the Indonesian banking sector, and the approaches to align and balance stakeholders’ claims according to theoretical perspectives and international best practices as found in the existing codes of corporate governance and international organizations. This sets the scene for examining the application of the stakeholder model in the Indonesian banking sector. This examination is pivotal in deciphering the relevance of the stakeholder approach in the Indonesian banking sector as well as a criticism of the dominance of the shareholder model in the industry so far. Finally, in the conclusion, a critique of three major flaws identified within the existing regulations is provided with recommendations for the next steps in the implementation of the stakeholder model that is compatible with the theoretical and best practice standards.

## Literature review

This section elaborates on two fundamental theoretical issues, namely the relevance of the stakeholder model and its concept and the application of the stakeholder model. There are two main issues addressed in the application of the stakeholder model, namely the principles used to identify the legitimate stakeholders, as well as align and balance the interests of stakeholders. It is expected that this theoretical assessment will provide a solid foundation for understanding the relevance of the stakeholder model, its concept, the basis for identifying parties who have a stake with a company (bank), and the alignment as well as balancing of stakeholders’ interests.

### Relevance of the stakeholder model and its concept

The relevance for both the creation and implementation of the stakeholder model of corporate governance is the belief that the shareholder model focuses too much on the welfare of shareholders to the detriment of other stakeholders [31]. While economists like Friedman [37] accept that managers are obliged to maximize shareholder interests, legal scholars and lawyers like Esser and du Plessis [33] and Anabtawi and Stout [1] do not agree, pointing out that directors and managers have duties to the company as a separate legal entity. However, there is a consensus among scholars, practitioners, and the judiciary that at the heart of the shareholder model is the idea that managers are obliged to maximize the shareholders’ interests [49, 52]. Sundaram and Inkpem [92] argue that the shareholder model is “the best among all available alternatives, and thus the preferred goal for managers formulating and implementing a strategy”.

Due to the fact that the shareholder model tends to focus solely on the welfare of shareholders (shareholder primacy), it has been then criticized as being too self-centric and disregarding ethical and moral issues [19]. Henry (2001, p. 161) calls this dilemma “moral egoism”, a situation where the principal’s natural ego heavily governs managers so that they act to serve only the principal’s own welfare. Consequently, other stakeholders can be subject to opportunistic exploitation by the firm’s managers and its shareholders, since professional managers are responsible only for the corporation’s stockholders’ welfare. In addition, smith [91] argues that the shareholder theory is “geared toward short-term profit maximization at the expense of the long run”.

For these reasons, the stakeholder model tries to resolve the shareholder model’s moral deficiency. Phillips, Freeman, and Wicks [44] convincingly stated that “the stakeholder theory is distinctive because it addresses morals and values explicitly as a central feature of managing organizations”. Jones and Wicks [59] also insist that the stakeholder approach has a normative legitimation that is “explicitly and unabashedly moral” and helps managers to conduct their business in an ethical way. Furthermore, Fassin [35] asserts that the stakeholder approach has become a critical instrument to transfer ethics to the management practice and strategy.

The stakeholder model, therefore, as pointed out by Freeman, Wicks, and Parmar [44] has two main tasks. First, it deals with the goals of the company. Professional managers are encouraged to articulate a shared sense of the value that they create, and what brings its core stakeholders together. Second, it focuses on professional managers’ responsibilities to their stakeholders. Professional managers are encouraged to articulate the way that they do business, in particular, the types of relationships that they want to build with their stakeholders to achieve their goals. Hence, Freeman and Reed [43] concluded that companies, in the long term, can survive if they are able to positively collaborate with all stakeholders in the network. According to Donaldson and Preston [29], in the stakeholder model of corporate governance the primary goal of firms is that professional managers should recognize the diversity of the stakeholder interests and properly devote attention to them in a mutually supportive framework. By doing so, professional managers then have a moral basis to function adequately. Post, Preston, and Sachs [81] firmly state that “these relationships are the essential assets that managers must manage, and they are the ultimate sources of organizational wealth”.

From the explanation above, there are several fundamental differences between the two models. The shareholder model itself is an approach that prioritizes the interests and welfare of shareholders alone, while it neglects the ethical and moral considerations in doing a business. Consequently, it may lead to the expropriation of the interests and welfare of non-shareholders. Contrary to that, the stakeholder model puts forward and balances the various interests and welfare of the stakeholders, including the shareholders themselves. Ethical and moral aspects are part of the business entity and at the same time become the guiding star for strategic and daily management decisions.

Furthermore, in the context of the banking industry, the stakeholder model, therefore, needs to be taken into account that legitimate stakeholders at the core of banks’ business matters. The Regulations on Corporate Governance in Indonesia should articulate a clear definition of stakeholders, how they can be identified, and what approaches can be utilized to align stakeholder claims. Under this perspective, both theoretical perspectives and international best practices can provide guidance on how to best achieve this, with adjustments for the Indonesian banking sector context.

Several guidelines with respect to the application of the stakeholder model can be found in the codes of corporate governance in jurisdictions such as the Netherlands and Germany. The stakeholder perspective has also been recognized by scholars and international organizations, such as UNCTAD, G20/OECD, and the Basel Committee for Banking Supervision. There are no international best practices that provide guidance specifically for the Indonesian banking sector. However, identifying the fundamental principles and best practices in relation to the application of the stakeholder model more generally can assist Indonesian lawmakers, more specifically the IFSA, to amend the Regulations on Corporate Governance to provide greater clarity and guidance on the issue of the stakeholder concept and its application.

Having both a clear definition of stakeholders and an understanding of how to identify legitimate stakeholders is critical for Indonesian lawmakers, more specifically the IFSA. For these reasons, it is important to review the degree to which the IFSA Regulations on Corporate Governance contain these features based on the theoretical perspectives and principles found in select codes in other jurisdictions, such as in the Netherlands and Germany as well as international organizations like UNCTAD, G20/OECD, and the Basel Committee.

Freeman [38] defines stakeholders as “any group or individual who can affect or is affected by the achievement of the firms’ objectives. This definition necessitates the identification of those who ‘can affect’ and those who ‘are affected’”. In Freeman’s opinion, those who “can affect” are stakeholders. Despite the critique that his definition is quite broad [58], Freeman’s views have been extensively accepted by scholars [48]. Freeman’s early conception of a stakeholder has been further developed by other scholars. Clarkson [22], for instance, conceptualizes stakeholders as “persons or groups that have or claim ownership, rights, or interests in a corporation and its activities, past, present, or future”. Donaldson and Preston [29] also sought to clarify the stakeholder concept. According to them, managers of a firm should also consider many interests such as those of the government, political groups, investors, employees, suppliers, customers, trade associations, and communities.

Freeman et al. [46] revised Freeman’s preliminary stakeholder model with the introduction of their two-tier stakeholder map. This map divides the stakeholders of a company into two categories. The first is internal stakeholders (the inner circle): employees, suppliers, financiers, customers, and communities. These groups define most companies’ business operations. Managers need to pay special attention to these groups and comprehend the values and purposes that are at stake here. The second is external stakeholders (the outer ring): competitors, consumer advocate groups, the government, the media, and special interest groups. These groups can affect, or be affected by, the company and influence the relationship between the company and its primary stakeholders.

The Dutch Corporate Governance Code of 2016 (the Dutch Code) and the Basel Committee for Banking Supervision Corporate Governance Principles for Banks (the Basel Committee Principles) contain best practice definitions and identify legitimate stakeholders. The Dutch Code [94] defines stakeholders as: “groups and individuals who, directly or indirectly, influence—or are influenced by—the attainment of the company’s objectives”. This code recognizes the following six categories of stakeholders. They are employees, shareholders, other lenders, suppliers, customers, and other stakeholders.

In the banking industry specifically, the Basel Committee Principles [12] do not provide a definition of stakeholders. However, they do identify four main legitimate stakeholders in the banking industry, such as depositors, shareholders, market participants, and other relevant or recognized stakeholders. However, the Basel Committee Principles [11] more clearly identify who other relevant or recognized stakeholders may be. These principles elaborate that due to the unique role of banks, the recognized stakeholders are varied across jurisdictions. They could, for example, be supervisors, the government, bondholders, and deposit guarantee institutions.

### Application of the stakeholder model: the principles used to identify and align stakeholders’ claims

Scholars have provided a number of theoretical approaches to align and weigh legitimate stakeholders’ claims. These approaches provide principles that can be used when taking into account a company’s stakeholders’ interests. The IFSA Regulations on Corporate Governance and Risk Management do not currently articulate such principles.

Opponents of the stakeholder model of corporate governance argue that the model has failed in several ways. For example, some say that the stakeholder model lacks accurate ethical guidance and the normative foundation needed by management to resolve diverse interests or conflicts of interest [13, 28, 30, 82]. Due to this perspective, [28] argues that the stakeholder model is vulnerable to Friedman’s view that the only goal of a business is to maximize shareholders’ value. To overcome the stakeholder model’s normative and moral foundation flaws, scholars have enriched the stakeholder model by providing numerous normative justifications [59, 13, 21, 22, 31, 36, 57, 100, 2]; Clarkson, 1994; Evan & Freeman, 1999; Phillips, 2002; Phillips and Reichart, 2000).

The debate on the alignment of the interests of stakeholders relies on the notion that interests should be considered by a company in its objectives and daily decisions. Scholars have been divided on the approaches to align stakeholder claims. Various approaches have been proposed to weigh stakeholder claims. However, all these approaches focus on the basis for legitimacy of the relationship. Cornell and Shapiro [23] argue that a party might have some claims to a company due to its implicit or explicit contract with the company. Implicit claims are not based on a written contract, while explicit claims are from those who have a clear written contract with the company. Donaldson and Preston [29] suggested that individuals are stakeholders in a company when they have an implicit or explicit contract with the company. However, they also advocate that individuals are acknowledged through “the real and potential harms and benefits they experience or anticipate experiencing as a result of the firm’s actions or inactions”.

Carroll and Buchholtz [18] believe that a party can have a stake in a company if it simply has moral and legal rights. An example of a moral right is the situation where workers who have been employed by a company for 30 years may assume that they possess the right not to be fired. Examples of legal rights include the right to fair treatment and the right to privacy. Hill and Jones [51] maintain that a party can have a stake in a company due to its critical resource contributions to that company. Based on this, whoever has made such a contribution can expect that their interests will be addressed properly by a company.

Still using the basis for legitimacy of the relationship framework, Evan and Freeman propounded (1998, pp. 75–76) that a party has a “stake in or claim on the firm” if it benefits from, or is harmed by, a company, or its rights are breached or satisfied by the company’s actions. If, for example, a party’s well-being is determined by the company, or they have moral and legal claims for a company as a result of the decisions to act or not to act, according to Langtry (1998), they are also stakeholders. When applying the basis for legitimacy of the relationship framework, a party can have a stake in a company for many reasons. These reasons can range from having implicit and explicit claims, possessing moral and legal rights, to even bearing the risks and deriving benefits. All these claims can be based on both unwritten and written contracts.

In addition to the above theoretical perspectives on approaches to weigh legitimate stakeholders’ claims, the importance of considering stakeholders’ interests is illustrated in codes of corporate governance in jurisdictions such as the Netherlands and Germany. Aligning stakeholders’ interests is also supported by international organizations such as G20/OECD and the Basel Committee for Banking Supervision.

In the German Corporate Governance Code (the German Code) (2017), the term “stakeholders” is used to highlight a management board’s responsibility to take into account the interests of the shareholders, its employees, and other stakeholders, with the objective of a sustainable creation of value. Whereas in the Dutch Corporate Governance Code (the Dutch Code) [94], the term “stakeholders” is deployed to signify that the existence of stakeholders should be recognized in a company’s long-term goals where those goals are based on the notion that “a company is a long-term alliance between the various stakeholders of the company”.

The Dutch Code [94] identifies three important stakeholder interests that should be aligned by a board. The first concern is the sustainability and long-term value creation of the company and its strategy. The second importance is balanced and effective decision-making and functioning of the board. The third attention is the protection of stakeholder interests in takeover situations. Consequently, stakeholders’ interests should be taken into consideration by boards. To do this, boards should be effective at entrepreneurship and efficient supervision, integrity, transparency, and accountability.

The Dutch Code also positions the shareholder as one of the company’s stakeholders. Does this mean that the interests of other stakeholders are equal to the interests of shareholders? To some extent, the Dutch Code still gives a degree of priority to shareholders to first secure their own interests (shareholder primacy) but without ignoring the interests of other stakeholders. The Dutch Code [94] provides that:

Shareholders can give priority to their own interests, as long as they act in keeping with the principles of reasonableness and fairness in relation to the company, its organs, and their fellow shareholders. This includes the willingness to engage with the company and fellow shareholders. The greater the interest which the shareholder has in a company, the greater is his responsibility to the company, fellow shareholders, and other stakeholders.

In the light of this perspective, how do boards, managers, and more broadly companies align the various interests of stakeholders? The Dutch Code [94] offers two fundamental principles that can be considered when aligning the various interests of stakeholders. Such action should be based on the principles of “reasonableness” and “fairness”.

The importance of aligning stakeholders’ interests is also supported by international organizations such as the G20/OECD Principles and the Basel Committee Principles. The G20/OECD Principles [95] highlight the importance of the role that stakeholders play in corporate governance mechanisms as well as the responsibility of the board to stakeholders. Regarding the role of stakeholders specifically in corporate governance, it is recommended that their role should be acknowledged when creating wealth, jobs, and the sustainability of financially sound enterprises. The principles also emphasize the importance of employees participating in the company.

Regarding the responsibility of the board, the G20/OECD Principles [95] suggest that boards are required to account fairly to stakeholder interests, including those of employees, creditors, customers, suppliers, and local communities. Boards should also apply high ethical standards by considering the interests of stakeholders, including when the board is developing the company’s code of conduct.

In the banking industry, the Basel Committee Principles [12] use the term “stakeholder” when describing, among other things, the formulation of corporate governance mechanisms, the board’s duties, disclosure requirements, and the need to be transparent. The Basel Committee Principles [12] state that “the primary objective of corporate governance should be safeguarding stakeholders’ interests in conformity with public interests on a sustainable basis. Among stakeholders, particularly with respect to retail banks, shareholders’ interests would be secondary to depositors’ interests”. The supervisory board and its senior management (the management board) should “protect the interests of depositors, meet shareholder obligations, and take into account the interests of other recognized stakeholders, and the legitimate interests of depositors, shareholders, and other relevant stakeholders” [12].

It is expected that the Indonesian banking sector should consider deploying the above experts’ opinions and best practices to balance the various interests of the stakeholders in the industry as the existing IFSA regulations do not cover this issue. Certainly, it is also desired that the IFSA should consider adopting some of these principles in its regulations on the application of the stakeholder model in the Indonesian banking sector.

## Research questions and methodology

This research has two main research questions. First, why is the stakeholder model relevant for the Indonesian banking sector? Second, what are the main flaws in the introduction of the stakeholder model in the existing IFSA regulations on corporate governance? In the second problem, there are three specific research questions to be addressed. They are (a) What should be the proper concept of stakeholders for the Indonesian banking sector? (b) Who should be the legitimate stakeholders of the Indonesian banking sectors? (c) How can the legitimate stakeholders of the Indonesian banking sector be aligned. This research applies a doctrinal legal research, which is a research approach that offers a systematic explanation of a particular legal category, provides a rules relationship analysis, explains difficult areas of the rules, and possibly predicts future developments (Pearce et al. 1987; Hutchinson and Duncan 2012).

In this research, legal primary documents such as the 2016 IFSA General Banks’ Corporate Governance Implementation Regulation and the 2015 IFSA PCBs’ Corporate Governance Implementation Regulation were the main sources of the research. From these two regulations, the existing definition of corporate governance and the goals of the corporate governance mechanism in the Indonesian banking sector were identified and analysed.

Other secondary sources such as international codes and/or guidelines on corporate governance such as the 2016 UK Code, the 2016 Dutch Code, the 2017 German Code, the 2015 OECD/G20 Corporate Governance Principles, the 2015 Basel Committee Corporate Governance Principles, and relevant journals were used to analyse and construct the proper definition of stakeholders, the legitimate stakeholders of the banking industry, and the alignment of the interests of stakeholders.

Based on Hutchinson and Duncan’s (2012) perspective, these various documents were analysed descriptively to determine what should be the essence of the above two ISFA regulations. This research strived to construct a proper definition of corporate governance and the appropriate goals of implementing corporate governance in the Indonesian banking sector.

## Results and discussion

### The stakeholder model in the Indonesian banking sector

#### Relevance of the stakeholder model for the Indonesian banking sector

In Indonesia, two sorts of banks are recognized, namely general banks (commercial banks) and People’s Credit Banks (PCBs) or the Indonesian banking sector. General banks and PCBs are conventionally run and based on Sharia principles. The difference, however, is that general banks provide payment transaction services, while PCBs do not (s 1 (2) (3) of the 1998 Banking Law). The main functions of both general banks and PCBs in Indonesia are to collect funds (savings) and distribute the funds (intermediary function, credits) (s 5 (1) of the 1998 Banking Law).

Theoretically, financial institutions like banks play a vital role in enhancing innovation and economic growth by funding economic, productive, and investment activities (Bagehot [5], Schumpeter 1912, in King and Levine 61]. Empirically, as indicated by Kind and Levine [61], the financial intermediation of banks could enhance the sustainability of economic growth, capital accumulation, and productivity. However, as revealed by some researchers [47, 15, 88] Rissy [85, 86] the Indonesian banking sector has suffered from severe crisis, especially in the 1999 crisis, due to mismanagement, corruption, internal fraud, poor corporate governance, and the application of a strong shareholder primacy culture. For these reasons, to reiterate, the introduction of stakeholder model by IFSA in the industry is a strategic step for the industry but the relevance of the introduction of the stakeholder model needs to be further examined.

To do this examination, there are three issues to be considered. The first issue is that historically, the Indonesian banking industry has been managed under a strong shareholder model. A lack of good corporate governance and risk management resulted in recklessness and in some cases, fraud on the part of general banks and ultimately a significant number of closures of general banks by Bank Indonesia during the 1999 financial crisis [20, 70, 83, 88] and later the closure of PCBs in 2010s by IFSA (Info Bank [54]). There were 61 general banks liquidated by Bank Indonesia and the Indonesian Banks Restructuring Agency during after the 1999 crisis [60]. These banks were mainly managed by shareholders or their affiliates or the government. The critical problem was that the Indonesian banking boards focused solely on securing the shareholders’ interests while neglecting and even expropriating other stakeholders’ interests. Meanwhile, in the PCBs case, Nelson Tampubulon, the Head Executive of Banking Supervision for the IFSA, said that 80% of PCB closures were caused by fraud [72] committed by management, including members of the board of commissioners (BOCs) or the supervisory board and board of directors (BODs) or the management board. Some of the managers and controlling shareholders committed fraud by misusing and embezzling depositors and lenders’ money, or by using the funds to further their personal interests (IFSA [54]).

While considering that fraud can occur where there is a culture of shareholder primacy, in the case of PCBs and general banks, management can act without due diligence and care when taking into account non-shareholders’ interests. However, this was not the only cause of failure in the sector. Factors such as poor internal audits and management’s lack of integrity also contributed to the fraud that took place (IFSA 54).

The second issue to be considered when examining the relevance of the stakeholder model for the Indonesian banking sector is that most banks are managed under a strong family firm-dominated culture. For example, as of August 2019 there were 1,745 PCBs across Indonesia which consisted of 1,581 (90.6%) conventional limited liability companies and 164 (9.4%) Syariah limited liability companies (IFSA [55]). Regardless of their legal entities, these banks are generally owned by the local government or by certain families. The majority of BOCs (supervisory board), BODs (management board), and key executive members are the banks’ shareholders, and their families and relatives or related parties are appointed by these shareholders (Rissy 2018). Thus, they are often a family-dominated firm (Miller and Miller 2003).

While it is argued that such a context can lead to lower leadership costs, social capital creation, an opportunistic entrepreneurship process [17], and the reduction of conflicts of interest [24], it can also lead to the expropriation of minority shareholders or the neglect of other stakeholders’ interests. This in turn can result in a conflict of interest between the principals and the majority and minority shareholders. It can also create a conflict of interest between shareholders and other stakeholders. Young et al. (2008) argue that a conflict of interest involving the controlling shareholders and minority shareholders is the result of concentrated ownership, massive family ownership and control, business group structure, and weak legal protection of minority shareholders. This situation can be exacerbated in emerging economies, where recourse to the courts when a BODs does not protect minority shareholders’ interests, is inadequate.

Historically, Indonesia’s banking sector has been managed in a way that has displayed a tendency to favour the shareholder primacy model. In practice, this has manifested itself in some members of BOCs and BODs and controlling shareholders misusing their positions to expropriate other stakeholders’ interests. Some of them wilfully plan and commit a fake credit scheme proposal. They then receive and use the money for their personal interests. In the case of general banks, during the 1999 financial crisis, the majority of the shareholders and board members was the main perpetrators of internal fraud and corruptions [20, 21, 83, 88].

In the case of PCBs, shareholders and members of boards marked up the amount of credit, embezzled depositors and customers’ money, and issued fake bank letters of guarantee (IFSA [54], 96–97, [6] Joglosemar [58]; Wartahukum [102]; [25]; Neracacoid 2016; IFSA 2015). Hence, the above disadvantages of a family-dominated firm—specifically the recklessness of management and the neglect of stakeholders’ interests (other than shareholders’ interests)—are identifiable problems. The “moral egoism” of the management and shareholders, as articulated by Henry (2001, p. 161) is a significant problem. There have been instances where the Indonesian banking sector management and its shareholders have intentionally exploited non-shareholders and stakeholders for their own selfish purposes.

Still, in the case of PCBs, another main issue to be considered when examining the relevance of the stakeholder model for PCBs is that as the majority of PCBs are family-dominated firms, the concentration of ownership and the unification of ownership and control are a reality. This can lead to the expropriation of minority shareholders and mismanagement. Theoretically, these issues could be mitigated by implementing the stakeholder model. Hence, the IFSA’s commitment to require PCBs to apply the stakeholder model is consistent with the suggestion proposed by scholars. Shleifer and Vishny (1997), for example, argue that the stakeholder model of corporate governance is more effective in accommodating conflicts in circumstances of concentrated ownership. Drawing upon Shleifer and Vishny’s ideas, in the Indonesian baking sector, the supervisory board can play a role to objectively prevent the use of a bank’s funds that can endanger the interests of minority shareholders, including scrutinizing the related parties’ transactions that can lead to the embezzlement of minority shareholder funds.

With an awareness that this problem continues to endanger PCBs’ business goals and their stakeholders’ interests, the IFSA has prohibited the majority of members sitting on a BOC and BOD from being family members of shareholders (ss 27–29 of the 2014 IFSA People’s Credit Banks Regulation). In an attempt to address the past practices, the IFSA acknowledged the importance of the stakeholder model for general banks (2016) and PCBs (2015) in the Regulations on Corporate Governance. This has been a strategic milestone in the creation of better and more effective management of the Indonesian banking sector.

In the context of the Indonesian banking sector, the application of the stakeholder model could mitigate the above three issues. When the management of general banks and PCBs works effectively under a stakeholder model, they are expected to consider and secure all stakeholders’ interests. The stakeholder model requires management to create value for all stakeholders [44] and recognize the diversity of stakeholders’ interests and respond to these interests within a mutually positive scenario [29]. However, it is important to emphasize that this can only happen if BOCs (supervisory board), BODs (management board), and other key executives alter their focus on the shareholder primacy model and start to consistently manage the Indonesian banking sector under a new stakeholder primacy model.

Banks’ management may also increase their effort to balance the various interests of stakeholders by publishing their financial reports transparently, so that parties that have a stake with banks such as debtors, creditors, and employees can assess the validity of the allocation and use of a bank’s funds, including the use of funds in the attainment of a bank’s business expansion. These steps are in line with Articles 31 (3) (4) and 60 of the IFSA Corporate Governance Regulation for General Banks of 2016 and Articles 29 (3) (4) and 66 of the IFSA Corporate Governance Regulation for PCBs of 2015 which stipulate that BODs should supervise related parties’ transactions in a bank and banks should publish their financial statements, including related parties’ transactions to the IFSA and to their stakeholders periodically. These steps might significantly encourage all stakeholders to increase their control to banks’ management when dealing with recognizing and balancing the different interests of stakeholders.

From a practical consideration, stakeholders can also unite, be active, and increase the pressure to relevant parties to accommodate their interests. In the case of a debt dispute between BDNI and IBRA, for example, it is known that when BDNI experienced liquidity distress due to financial distress in the 1999 crisis, BDNI received IDR 4.8 trillion in liquidity aid from Bank Indonesia. The liquidity aid was calculated as debt that had to be repaid to Bank Indonesia (later BRA). However, BDNI was only able to return IDR 1.1 trillion. This means that BDNI owed IDR 3.7 trillion in debt to IBRA. During the restructuring process, it was revealed that some of the funds had been used by BDNI’s previous management to pay off the debt of another private party, namely the PT Gajah Tunggal group. Therefore, the remaining debt should be billed by IBRA to PT Gajah Tunggal Group and the controlling shareholders. Facing this problem, BDNI direct stakeholders such as BDNI’s CEO, shareholders, and employees united and carried out an active lobby and pressured IBRA to release BDNI from the remaining debt. IBRA then issued a release and discharge letter to BDNI [68] (Tamenggung 2019).

In addition, the stakeholder social pressure approach could also be deployed to change a company’s management attitude. In a research conducted by Rudyanto and Siregar [87] on 123 Indonesian listed companies’ sustainability reports, it was revealed that social pressure, especially from the community and the environment around the company and consumers, could actually make companies improve the quality of their sustainability reports.

It is expected that under a stakeholder model which is supported by sound corporate governance standards, BOCs (supervisory board), BODs (management board), and other key executives of the Indonesian banking sectors will be able to manage the industry more effectively by taking into account and balancing all stakeholders’ interests, and by contributing to the sustainability of the Indonesian banking sector.

#### Weaknesses of the current regulations on the stakeholder model in the Indonesian banking sector and their solutions

As previously indicated, the IFSA Regulations on Corporate Governance only provide a broad definition of the term “stakeholders”. Stakeholders are simply defined as all parties that directly or indirectly have interests with a bank’s business operations (s 1 (8) of the 2015 IFSA Corporate Governance Regulation for PCBs and s (1) of the 2016 IFSA Corporate Governance Regulation for General Banks). The regulations do not clearly clarify which parties have direct and indirect interests in PCBs and general banks. Nor do they identify general banks and PCBs’ legitimate stakeholders, and how to properly align the industry stakeholders’ interests. In this section, the author will explore these weaknesses and provide solutions to overcome the identified flaws.

The definition of a stakeholder is found in s 1 (8) of the 2015 IFSA Regulation concerning Corporate Governance for PCBs and s 1 (8) of the 2016 IFSA’s Regulation concerning Corporate Governance for General Banks. A “stakeholder” is defined as all parties that directly or indirectly have interests with a PCB’s business operations. Unfortunately, there is no further explanation regarding who can be categorized as “directly” or “indirectly” having interests with PCBs. This broad definition is similar to those given by Freeman and the Dutch Code. Included in Freeman’s definition [29] are the words “who can affect or is affected” and the Dutch Code provides for (2016) those “who directly or indirectly influence or are influenced”.

The difference, however, is that the IFSA’s Regulations on Corporate Governance do not go further and identify the legitimate stakeholders in the Indonesian banking sector; parties that have interests “directly” or “indirectly” with the Indonesian banking sector. Freeman [38] and the Dutch Code [94] clearly establish those parties who can “affect” or are “affected” by the achievement of the firm’s goals and those “who directly or indirectly, influence—or are influenced by the achievement of the company goals”. Freeman [38] identifies five internal stakeholders of a company such as employees, suppliers, financiers, customers, and communities. Fassin [35] recognizes six external stakeholders of a company. They are non-governmental organizations, environmentalists, the government, the media, critics, and others. In comparison, the Dutch Code [94] also identifies six stakeholders like employees, shareholders, other lenders, suppliers, customers, and other stakeholders. Additionally, in the banking industry, the Basel Committee Principles [12] identify four categories of stakeholders like depositors, shareholders, market participants, and other relevant or recognized stakeholders that are recognized by law, such as the supervisory institution, the government, bondholders, depositors, and the deposit guarantee institution.

**Fig. 1:**
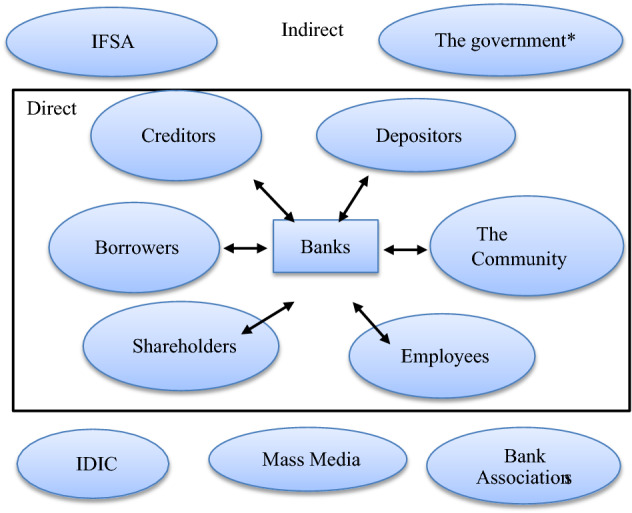
The Indonesian Banking Sector Legitimate Stakeholder. * the government can also be a direct legitimate stakeholder of state-owned banks

Drawing on the above for guidance for general banks and PCBs, the IFSA Regulations on Corporate Governance should be amended to include a clear definition of ‘direct’ stakeholders. This definition should include six parties for a bank: (1) borrowers: parties who borrow money to finance their business and other consumption activities; (2) depositors: parties who save their money in banks; (3) creditors: parties who lend their money to banks; (4) shareholders; (5) employees; and (6) the community: people who live near the banks. A definition of banks’ “indirect” stakeholders should also be included in the regulations and comprise: (1) the government; (2) the regulator/supervisor: the IFSA; (3) the deposit guarantor: the Indonesian Deposit Insurance Cooperation (IDIC); (4) the mass media; and (5) bank associations. Figure 1 illustrates recommendations for how “stakeholders” should be identified and defined in the two IFSA Regulations concerning Corporate Governance.

Therefore, for the Indonesian banking sector, stakeholders should be defined as all parties that directly or indirectly have interests with the Indonesian business operation. Parties that have a direct stake with a bank are those in which their involvement directly determines the long-term success of a bank. They are depositors, creditors, shareholders, employees, the community, and the government (for state-owned banks). Meanwhile, parties that have an indirect stake with a bank are those in which their involvement may determine the long-term success of a bank. They are IDIC, IFSA, the mass media, bank associations, and the government (for private banks). For example, depositors can negatively affect the operations of banks if their interests are not properly aligned. In July 2020, there was a rush at seven major banks in Indonesia. The problem began when there was a massive depositors withdraw leading to a bank “run” [3] which was triggered by concerns about financial distress due to the COVID-19 pandemic. The banks explained that depositors’ money is safe, but it was not trusted by the depositors. As a result, these banks suffered from liquidity distress which could lead to the revocation of the banks’ business operations. IFSA then convinced the depositors that their funds were secured so there was no need to withdraw large amounts of funds as it could disrupt the banks’ business operations [63]. In this case, it becomes clear that depositors can determine the continuation of bank business operations when they believe that their interests are properly accommodated and secured by banks’ management.

The above approach to balance the interests of depositors carried out by the banks and the IFSA is in line with the suggestions highlighted by the German Code (2017) and the Dutch Code (2017) where the protection of the interests of stakeholders (in this case depositors) ultimately helps banks to achieve their long-term success or long-term value creation.

The Indonesian banking industry should owe all their direct stakeholders moral and legal obligations as their support, perspectives, and actions can significantly contribute to the continuation of their business. As proposed by the G20/OECD (2015), the Indonesian banking sector should balance the interests of the stakeholders by providing more opportunities to the stakeholders to participate in the decision-making process that aims to achieve wealth and job creation, greater accountability and transparency, and financial soundness. The supervisory board and its senior management (the management board) should also secure and balance the interests of its stakeholders by addressing the competing interests propositionally (the Basel Committee Principles, 2015), rationally and reasonably (the Dutch Code 2017).

These obligations should also extend to banks’ indirect stakeholders as their perspectives and actions can either benefit or significantly determine the continuation of a bank’s business and their internal legitimate stakeholders’ interests. By clearly defining and identifying banks’ legitimate stakeholders in the Regulations on Corporate Governance, IFSA can assist banks to address their legitimate stakeholders’ interests more effectively. Doing this may also strengthen the application of the stakeholder model of corporate governance in the Indonesian banking sector.

In addition, another vital issue that needs to be addressed in the Regulations on Corporate Governance is how the interests of stakeholders can be aligned in the Indonesian banking sector. To deal with this issue, the IFSA Regulations on Corporate Governance both for general banks (2016) and PCBs (2015) do not currently articulate principles that can be used to align bank stakeholders’ interests. As previously explained, scholars have provided approaches to weigh legitimate stakeholders’ claims. These principles can be used when taking into account a company’s stakeholders. It is recommended that they are incorporated into the Regulations on Corporate Governance. For example, the principles include that a party can have a stake in a company if it has an implicit or explicit contract with the company [23], or if it could experience real or potential harm due to the company’s actions or inactions [29]. Furthermore, a party can have a stake in a company if its rights can be either breached or satisfied by the company’s actions [34], or it makes critical resource contributions to the company [51] and has moral and legal rights in relation to the company [15].

International best practices also provide perspectives on how to address stakeholders’ interests that could be incorporated into the Regulations on Corporate Governance. The Dutch Code [94] provides two fundamental principles to be considered in aligning various interests of stakeholders: the “reasonableness” and “fairness” principles. To implement the “reasonableness” and “fairness” principles, the Indonesian banking sector should carefully scrutinize the issues they face with its stakeholders and try to balance the interests of various stakeholders where they conflict. For example, management might trade off the interests of communities against employees. Less probably, management might recognize and balance the interests of shareholders against the interests of bank employees when dealing with higher wages or better working conditions.

In addition, the Basel Committee Principles [12] recommend that the interests of stakeholders should align with public interests. It states that “the primary objective of corporate governance should be safeguarding stakeholders’ interests in conformity with public interests on a sustainable basis. Among stakeholders, particularly with respect to retail banks, shareholders’ interests would be secondary to depositors’ interests”. Under this perspective, when the management of a bank has to choose between increasing shareholder profits and repaying a bank’s obligations for its depositors, the option should be to pay back the bank’s obligations to the depositors. This option can increase the trust of depositors to the bank, which in turn can increase depositors’ support for the continuation of the bank’s business operations.

Given that there is no single principle to be used when considering stakeholders’ interests, it is recommended that the principles to be deployed should consider the characteristics of a particular company’s business, its background, and the environment, including the community, the political and social climate surrounding the company, and the stakeholder culture within the company. For this reason, it is critical, however, to acknowledge that whatever methods are deployed to align the various interests of stakeholders, there are two main realistic principles proposed by the author to be considered, namely normative and practical principles.

In the context of the Indonesian banking sector, it is suggested that revised regulations should clearly outline that IFSA requires the Indonesian banking sector’s BODs, BOCs, and other key executives to properly recognize and balance their stakeholders’ interests. Such recognitions and balances could be based on both normative and practical principles. Normative principles include idealistic and abstract principles such as moral and fairness values and rights that banks should be obliged to afford their stakeholders. Practical principles focus on pragmatic matters such as written contracts to which stakeholders and banks are party, the risks borne by a party or the benefits received by a party due to a bank’s activities, the real contributions that a stakeholder may make to a bank, and the relationship that a stakeholder has with a bank. It is critical that these principles are incorporated into the Regulations on Corporate Governance, so that they can be utilized to protect depositors’ money and avoid losses being incurred by depositors, especially if there is the closure and liquidation of a bank.

## Conclusion

The stakeholder model has been advocated and developed over the last thirty years. It is often acknowledged but its actual implementation in the context of the Indonesian bank industry raises several important issues that have been examined in this study. The stakeholder model is an appropriate governance model that has been introduced by IFSA for the Indonesian banking sector and should be implemented in the banks given that they have experienced various internal fraud, corruption, and mismanagement. These problems lead to the expropriation of banks’ non-shareholder stakeholders’ interests, such as employees, the community, lenders and depositors, and the closures of some banks.

The current IFSA Regulations concerning Corporate Governance for general banks (2016) and PCBs (2015) in dealing with the stakeholder model contain three key weaknesses. They are the vague definition of a stakeholder, the lack of guidance on how legitimate stakeholders may be classified, and the absence of guiding principles in the regulations that can assist with balancing stakeholders’ interests.

It is recommended that IFSA should properly explain the relevance of the stakeholder model for the Indonesian banking sector, concisely define the concept of a “stakeholder”, and clearly articulate which parties have direct or indirect interests in the industry. IFSA should consider the definition of a stakeholder proposed; that is all parties that directly or indirectly have interests or stakes with Indonesian Banking business operations. Parties that have a direct stake with a bank are those in which their involvement directly determines the long-term success of a bank. They are depositors, creditors, shareholders, employees, the community, and the government (for state-owned banks), while parties that have an indirect stake with a bank are those in which their involvement may determine the long-term success of a bank. They are IDIC, IFSA, the mass media, and bank associations, and the government (for private banks).

It is also suggested that IFSA should require BODs (supervisory board), BOCs (management board), and other key executives of the Indonesian banking sector) to properly, rationally, and reasonably consider or balance their stakeholders’ interests. To do so, the Indonesian bank sector should approach their stakeholders’ interests based on a normative principle, an approach that is based on ideal values such as fairness, ethics and morals, and a practical principle, an approach that relies on real considerations such as risks, contributions, benefits, and relationships.

## Limitations and future research

This study applied a doctrinal legal research methodology which predominantly relied on the understanding and interpretation of the author of the legal sources examined. Therefore, the results and discussions of this issue could be different for other authors. However, when the understanding and interpretation of this theme are based on the same succinct doctrinal legal research and theoretical background, the results and discussions might be similar. In the future, research should focus on the implementation of aligning legitimate stakeholders of the Indonesian banking sector.
